# A moveable surface coil fabrication for MR-guided HIFU ablation

**DOI:** 10.1186/2050-5736-2-S1-A20

**Published:** 2014-12-10

**Authors:** San-Chao Hwang, Hsu Chang, Ih-Yuan Kuo, Pai-Hsun He

**Affiliations:** 1 Division of Medical Engineering Research, National Health Research Institutes, Zhunan, Miaoli, Taiwan

## Background

In this study, a moveable MRI receive-only surface coil was fabricated and used to provide higher SNR MRI images focused on a small area. The moveable coil can be moved together with the HIFU transducer to improve the accuracy of MRI temperature measurement of HIFU ablation in a mini-pig model.

## Materials and methods

A movable circle surface coils with a low input impedance preamplifier were made to connect the receive system of a 1.5T MRI scanner (Symphony, Siemens). The coil was moved together with the HIFU transducer during the HIFU ablation. The dynamic temperature images during HIFU ablation of porcine meat and living mini-pig leg were measured by this home-made coil. MR temperature measurements were calculated based on proton resonance frequency (PRF) shift method. A spoiled gradient echo sequence was applied for temperature mapping (TR = 13ms, TE = 7 ms, flip angle = 30 deg, matrix 128x128, FOV= 256 x 256 mm, slice thickness = 8 mm).

## Results

The SNR ratio using our coil was improved at least 3 times than using the commercial spinal coil array for the case of pig leg HIFU ablation since the coil is more closely to the target. The standard deviations of temperature measured by the moveable surface coils were 0.3, 0.6 deg C for porcine meat and pig leg, respectively.

## Conclusions

The results of our HIFU studies showed that the MRI image signal-to-noise ratio was improved and thus the higher precision of the temperature measurement could be obtained. It showed the advantage and feasibility of using a smaller and mobile surface coil for MR-guided HIFU ablation.

